# Obituary: Mitchell P. Fink

**DOI:** 10.1186/s13054-015-1169-1

**Published:** 2015-12-23

**Authors:** Derek C. Angus

**Affiliations:** Department of Critical Care Medicine, University of Pittsburgh, 3550 Terrace Street, 614 Scaife Hall, Pittsburgh, PA 15261 USA

**Fig. 1:**
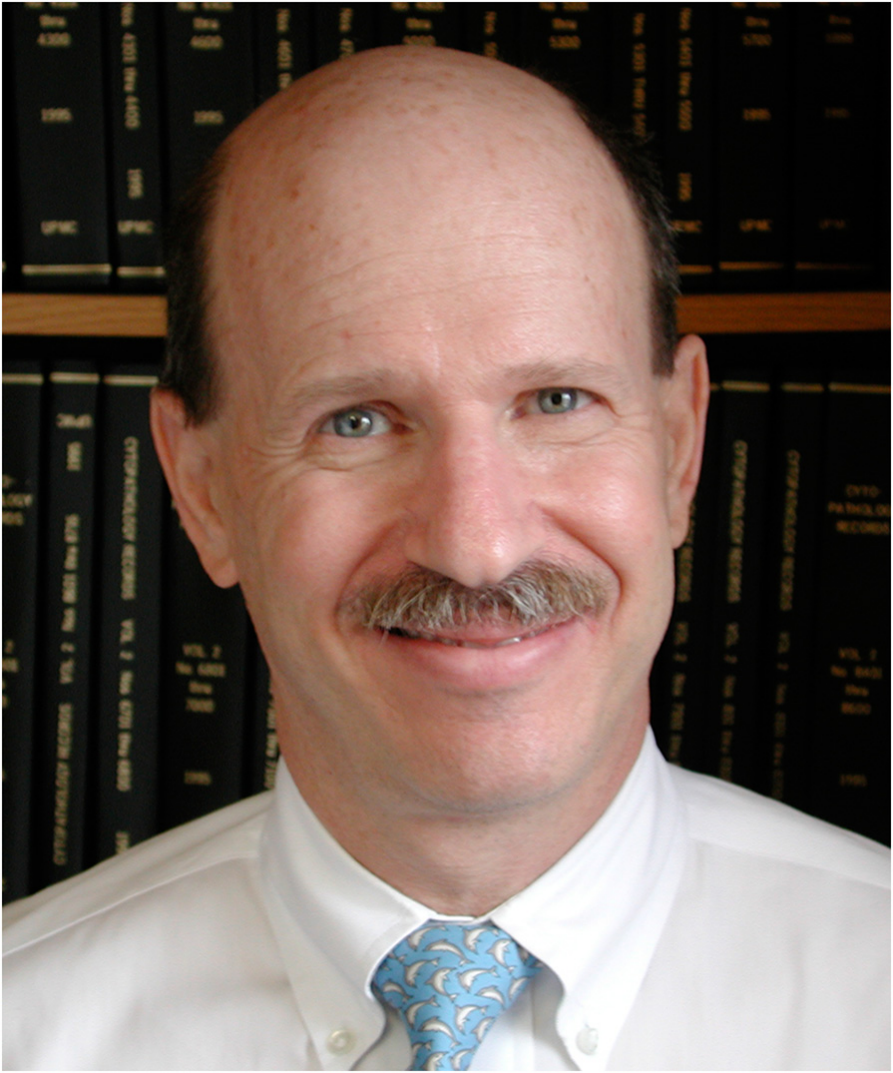
Mitchell P. Fink

Mitchell P. Fink, MD (Fig. 1), one of the most inspiring and influential leaders in the field of intensive care medicine, died at 66 years of age on 17 November 2015 after being diagnosed this summer with an aggressive sarcoma. This devastating news has left many of us with a huge sense of loss and sadness.

Mitch graduated from the Washington University School of Medicine in St. Louis, MO, in 1976 and completed general surgery training at the National Naval Medical Center in Bethesda, MD, in 1981. Thereafter, he held faculty positions at the Bethesda Naval Center and the University of Massachusetts, where he worked as surgeon and intensivist. In 1992 he moved to the Harvard Medical School, eventually becoming the Johnson and Johnson Professor in Surgery and surgeon-in-chief at the Beth Israel Deaconess Medical Center. He moved to the University of Pittsburgh in 1999, where he subsequently became the inaugural chair of the Department of Critical Care Medicine. He founded several companies based on discoveries from his laboratory and, in 2007, left the university to oversee the formation of one of these ventures. In 2009 Mitch returned to academia, joining the faculty at University of California Los Angeles where he held the title of Professor of Surgery and Vice Chair for Critical Care.

During his career, Mitch cared for thousands of patients with skill and compassion, amassed a great body of experimental and clinical research, trained and mentored hundreds of physicians and scientists, demonstrated wonderful entrepreneurship, and carved a vision of modern multidisciplinary critical care with lasting benefits for our entire field. For decades, academic medicine has praised the rare individual who excels as the so-called triple threat: teacher, clinician, and scientist. Mitch was all three and more, adding to these triple roles the rare gift of visionary leader in both academia and industry. I have never known anyone quite like Mitch.

His extraordinary career began early with a first author paper in *Nature* while still in medical school. While working in Bethesda, he forged close relationships with the National Institutes of Health critical care group, conducting a number of seminal experimental studies that both helped to establish the utility of animal models to probe the host response to sepsis, trauma, and shock and to inform our current understanding of these syndromes. Later, Mitch would conduct pioneering work using cell-based models of organ dysfunction to tease out the cellular response to stress from hypoxia and endogenous and exogenous danger signals, exploring key concepts of mitochondrial dysfunction and dysoxia. In addition to his passion for experimental research, Mitch also conducted many highly cited clinical studies, co-wrote the *Textbook of Critical Care*, and served on the editorial boards of numerous journals including *Critical Care*, *Critical Care Medicine*, and *Intensive Care Medicine*.

To all of his work, Mitch brought four enduring characteristics: knowledge, logic, creativity, and focus. He maintained an encyclopedic knowledge of the world, able to quote relevant papers, data, and information off the top of his head from a dizzyingly wide range of art, science, politics, and history. He also exercised crystal-clear logic, distilling the most complex problem into a series of simple steps, whether this was a complicated piece of basic science or a tough business decision facing a start-up biotechnology company. Having concisely summarized any problem, he then brought great creativity and inventiveness to probe and tackle its solution. Finally, he always kept a clear sense of purpose, making sure neither he nor his colleagues ever lost sight of the larger goals of advancing medical knowledge and saving lives.

I came to know Mitch during his tenure in Pittsburgh when he oversaw one of the most prolific and successful eras in our history, serving as a wonderful mentor and colleague, and launching the first multidisciplinary department of critical care medicine in a US medical school. In our daily contact with Mitch, we saw not only his intellect and drive, but also his warmth, compassion, wit, and grace. His door was always open, ready to listen to everyone from senior colleagues to junior residents and new staff. He had words of kindness and advice to coach us through every disappointment and words of praise and respect for every accomplishment. For me, as for many, Mitch was unquestionably one of the most supportive and influential people we have ever known. Through both his words and actions, he taught us not just about medicine and science, but about life, relationships, honor, duty, and service. We have lost an immense presence. Mitch is survived by his wife, Judy, and children, Matt and Emily. We extend our deepest condolences to them. He will be deeply missed.

